# miRwayDB: a database for experimentally validated microRNA-pathway associations in pathophysiological conditions

**DOI:** 10.1093/database/bay023

**Published:** 2018-02-28

**Authors:** Sankha Subhra Das, Pritam Saha, Nishant Chakravorty

**Affiliations:** 1 School of Medical Science and Technology, Indian Institute of Technology, Kharagpur, West Bengal, 721302, India; 2 Cryogenic Engineering Centre, Indian Institute of Technology, Kharagpur, West Bengal, 721302, India

## Abstract

MicroRNAs (miRNAs) are well-known as key regulators of diverse biological pathways. A series of experimental evidences have shown that abnormal miRNA expression profiles are responsible for various pathophysiological conditions by modulating genes in disease associated pathways. In spite of the rapid increase in research data confirming such associations, scientists still do not have access to a consolidated database offering these miRNA-pathway association details for critical diseases. We have developed miRwayDB, a database providing comprehensive information of experimentally validated miRNA-pathway associations in various pathophysiological conditions utilizing data collected from published literature. To the best of our knowledge, it is the first database that provides information about experimentally validated miRNA mediated pathway dysregulation as seen specifically in critical human diseases and hence indicative of a cause-and-effect relationship in most cases. The current version of miRwayDB collects an exhaustive list of miRNA-pathway association entries for 76 critical disease conditions by reviewing 663 published articles. Each database entry contains complete information on the name of the pathophysiological condition, associated miRNA(s), experimental sample type(s), regulation pattern (up/down) of miRNA, pathway association(s), targeted member of dysregulated pathway(s) and a brief description. In addition, miRwayDB provides miRNA, gene and pathway score to evaluate the role of a miRNA regulated pathways in various pathophysiological conditions. The database can also be used for other biomedical approaches such as validation of computational analysis, integrated analysis and prediction of computational model. It also offers a submission page to submit novel data from recently published studies. We believe that miRwayDB will be a useful tool for miRNA research community.

**Database URL**: http://www.mirway.iitkgp.ac.in

## Introduction

MicroRNAs (miRNAs) are small (∼22 nt), endogenous and non-coding RNAs which regulate gene expression at post-transcriptional level (1). They generally bind to seed-matched sites in 3′ untranslated regions (3′ UTRs) of their target mRNA(s) to cause translational repression. miRNAs have been shown to play a crucial role in majority of the known biological processes, such as development, differentiation and cell death (2). A number of studies have reported aberrant miRNA expression profiles as the hallmark of pathophysiological derangements leading to disease conditions by altering genes in molecular signaling pathway(s) (3–5). It is evident that identification of the experimentally validated miRNA-pathway associations will benefit researchers immensely to correlate them with pathophysiological derangements. However, sifting through the enormous association studies available is a daunting task abhorred by many.

To date, several bioinformatics resources, such as miRNA databases and computational tools have been developed. miRBase is the largest web-accessible database of all published miRNA sequences and annotation (6). Although researchers have primarily focused on miRNA databases for animals, databases also exist for other organisms for example, VIRmiRNA for viruses (7) and PMRD for plants (8). Computational tools and databases, such as TargetScan (9), miRanda (10), PicTar (11), RNA22 (12), PITA (13), miRecords (14), miRTarBase (15), miRTrail (16) and miRnalyze (17) have been designed to address the intricate relationship between miRNA and their mRNA targets. Some of the resources, for example, miTalos (18), DIANA-miRPath v.3 (19–21), MiRSEA (22), Subpathway-GMir (23) and miRPathDB (24) are focused on miRNA-pathway associations. However, the major limitation with such tools (except DIANA-miRPath v.3 and miRPathDB) is that they are mostly based on computational predictions and probabilistic models that often falter when investigated experimentally.

In order to address the issue of ‘translational paresis’ from theory to practical, researchers have begun focusing on the development of databases collating information on miRNA and disease associations (25). The HMDD database (built in 2007, last updated 2014) was the first miRNA-disease association database (26, 27). Another database, miR2Disease is a resource for experimentally validated miRNA dysregulation in various human diseases (28). PhenomiR provides information about differentially expressed miRNAs in diseases and biological processes (29). miRPathDB is the resource for miRNA regulated pathways (24). Unfortunately, to the best of our knowledge, there is no known database that can offer complete information about pathway regulation by miRNA(s) in a disease/pathophysiological condition. In this context, a database for experimentally validated miRNA-pathway associations in various pathophysiological conditions is expected to serve as an invaluable tool for the scientific community. Here, we have developed a manually curated miRwayDB database which provides information about the effect of miRNA expression on dysregulation of molecular pathways as observed in various disease conditions.

## Materials and methods

### Data search

The association data of miRNA and pathway in different disease/pathophysiological conditions were extracted from PubMed search engine [https://www.ncbi.nlm.nih.gov/pubmed/ (16 December 2017, date last accessed)]. The search term used was ‘(name of pathophysiological condition) AND (microRNA OR miRNA OR miR) AND (pathway OR signal transduction OR signaling network)’. For example, in case of breast cancer, we used search term: ‘(breast cancer) AND (microRNA OR miRNA OR miR) AND (pathway OR signal transduction OR signaling network)’. These lists were cross referenced and the duplications of published studies were removed. After exhaustive manual screening, only those articles that explored the effects of miRNAs in pathway dysregulation by targeting genes in different diseases were curated. Predictive studies that were not back by experimental validation for miRNA-pathway interactions in disease condition were removed. We focused on various human diseases which are caused due to miRNA mediated pathway dysregulation. Therefore, we restricted our database within the limit of human only.

### Database contents

**Figure 1.**
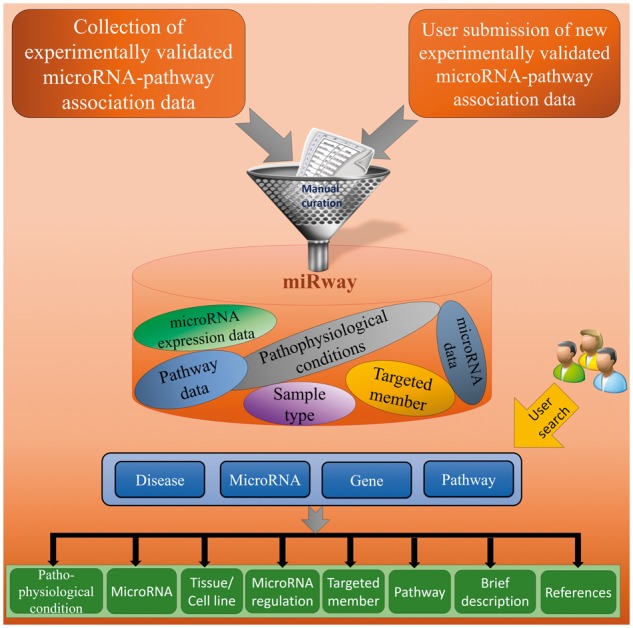
The schematic representation of miRwayDB.

miRwayDB is designed to ease discovery and searching for various disease related miRNAs, genes and pathways. Presently, miRwayDB records 663 miRNA-pathway associations between 232 miRNAs and 122 pathways in 76 human diseases. All the information in miRwayDB is collected from published studies and each miRwayDB entry is hyperlinked with PubMed. Each miRNA is further hyperlinked with miRBase (6) which is the primary repository for miRNA sequence and annotation. For annotation of pathways, we followed Kyoto Encyclopedia of Genes and Genomes (KEGG) pathway database (30, 31). Although, miRwayDB provides some novel miRNA-mediated pathway data which are exist in literature only. Tissues and cell lines are annotated using Brenda Tissue Ontology (BTO) (32) and Cellosaurus [https://web.expasy.org/cellosaurus/ (16 December 2017, date last accessed)] (Amos Bairoch, Swiss Institute of Bioinformatics) respectively. The schematic representation of database is illustrated in Figure 1.

### Database designing

The frontend of miRwayDB has been developed using HTML5. The server side of the database is supported by Laravel, a PHP framework which works on Model-View-Controller (MVC), an architectural pattern. miRwayDB is being managed by MySQL, a relational database management system.

## Results

### Web interface

**Figure 2.**
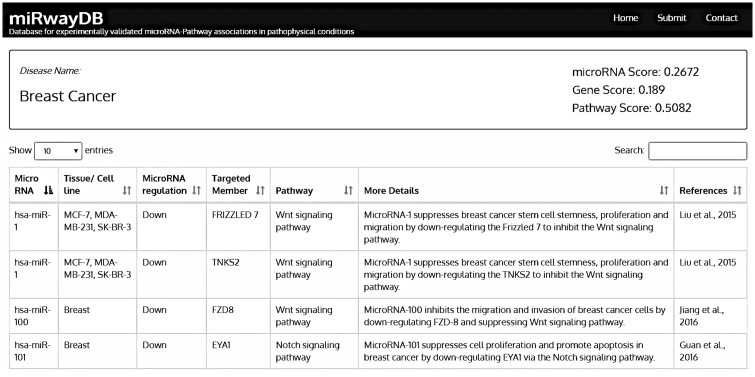
Search output of miRwayDB.

The database search page offers four separate search options for users: disease, miRNA, gene and pathway. The homepage also has a list of available pathophysiological conditions with hyperlinks to the miRNA-pathway associations in that condition. Each condition is further subdivided into a number of disease conditions. A user-initiated search will lead to the list of following parameters: disease name, miRNA name, tissue/cell line name, miRNA regulation (up/down), targeted gene, dysregulated pathway, brief description and reference (see Figure 2). User can sort the parameters by any column. The database also provides a submission page to submit new data from recently published studies. Once approved by our review team, the submitted records will be shown in the database. Such user initiated submissions will be updated immediately.

### Database statistics

**Figure 3.**
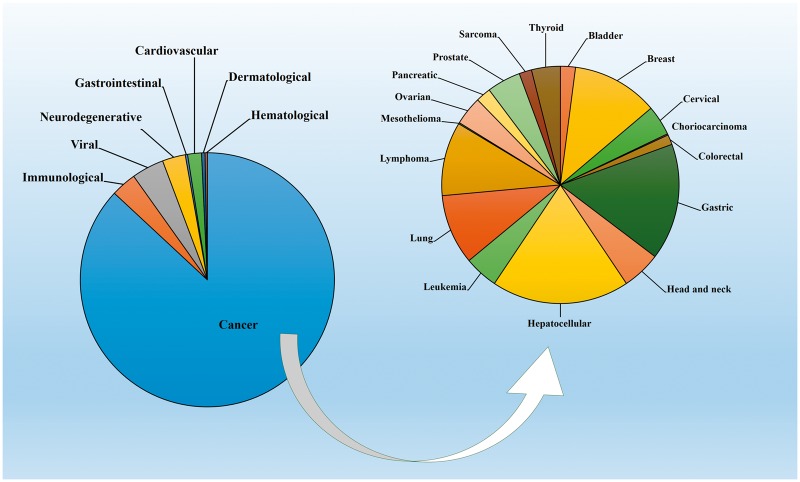
Distribution of pathophysiological conditions in miRwayDB.

miRwayDB is the publicly available resource of miRNA mediated pathway dysregulation in various pathophysiological conditions. miRwayDB datasets revealed that cancers (87%) are the most thoroughly studied diseases followed by viral diseases (4.11%), immunological diseases (3.23%) and neurodegenerative diseases (2.94%). Among the various cancer types, most of the studies have been done on hepatocellular carcinoma (18.58%) followed by gastric cancer (15.7%) and breast cancer (11.65%). Distribution of diseases in miRwayDB is presented in Figure 3.

**Figure 4.**
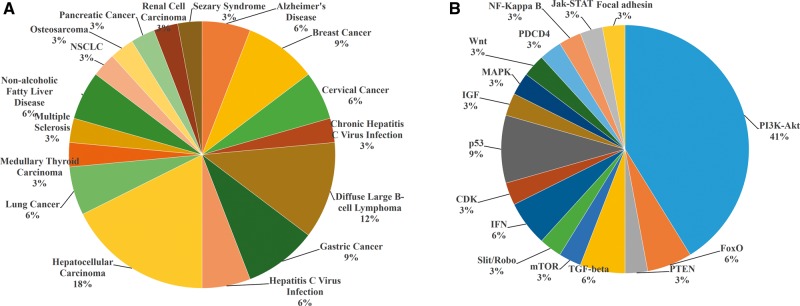
MicroRNA-21 associated pathophysiological conditions (**A**) and pathways (**B**).

Currently, miRwayDB collects an exhaustive list of miRNA-pathway association entries including 232 miRNAs and 122 pathways. Among the 232 miRNAs, the largest number of studies has been performed on miRNA-21 which is seen to be associated with 17 different pathophysiological conditions (Figure 4A). Among theses, hepatocellular carcinoma is seen as the most affected disease (18%) followed by diffuse large B-cell lymphoma (12%) and gastric cancer (9%). Dataset also reveals that deregulation of miR-21 altered 16 pathways (Figure 4B). PI3K-Akt signaling pathway is the most altered miR-21 targeted pathway (41%).

**Figure 5.**
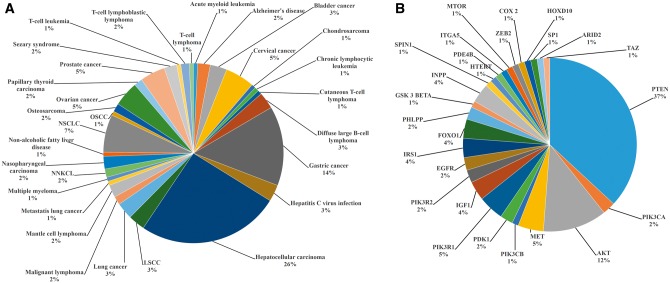
PI3K-Akt signaling pathway associated pathophysiological conditions (**A**) and dysregulated genes (**B**).

Survey of the pathway data shows that PI3K-Akt signaling pathway is the most thoroughly investigated pathway which is associated with 30 pathophysiological condition. PI3K-Akt signaling pathway associated pathophysiological conditions are illustrated in Figure 5A. PI3K-Akt signaling pathway genes which are altered due to dysregulation of miRNA expression are shown in Figure 5B.

### miRNA score, gene score and pathway score

**Figure 6.**
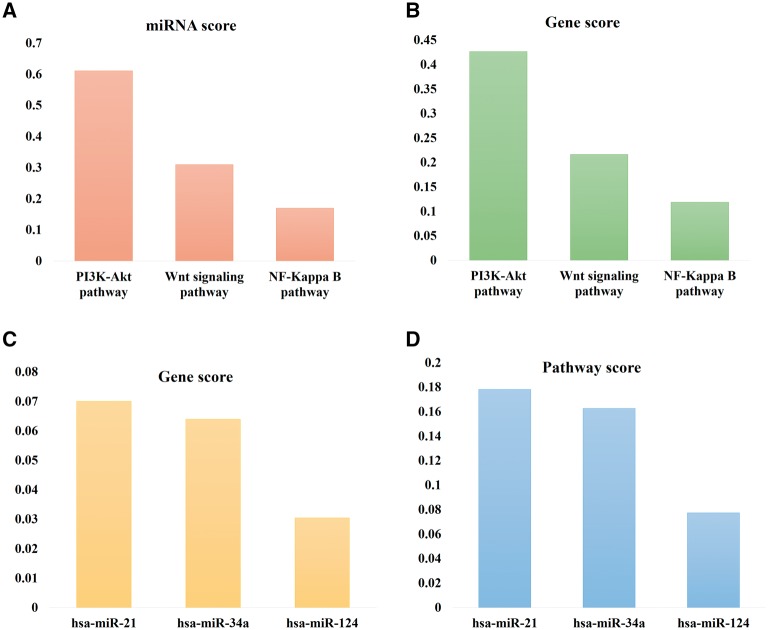
Top 3 pathways with highest miRNA scores (**A**) and gene scores (**B**); and top 3 miRNAs with highest gene scores (**C**) and pathway scores (**D**).

In order to assess the importance of miRNA mediated pathway alteration in disease, we have introduced three different scores, that is, miRNA, gene and pathway score. miRNA score of a pathway is defined as the fraction of dysregulated miRNAs altered a given pathway compared with total number of dysregulated miRNAs that have been reported to be associated with pathway alteration. A high miRNA score of a pathway is therefore a reflection of a large number of studies confirming several miRNAs targeting that pathway. This in turn is indicative of the growing interest about that particular pathway in the research community and hence suggestive of its relative importance especially clinical since we are correlating them with critical diseases/pathophysiological conditions. This score can be used to evaluate the importance of a miRNA regulated pathway in various pathophysiological conditions. For example, PI3K-Akt signaling pathway (Figure 6) has the highest miRNA score (0.6207) suggesting that this pathway genes are targeted by most number of miRNAs and miRNA mediated PI3K-Akt signaling pathway alteration is responsible for many disease. Earlier (Figure 5), miRwayDB data showed that PI3K-Akt signaling pathway plays significant role in 30 pathophysiological conditions. Using similar concept, we introduced gene score of miRNA, pathway and disease; and pathway score of miRNA, gene and disease. Top 3 pathways with highest miRNA and gene scores; and top 3 miRNAs with highest gene and pathway scores are listed in Figure 6. MicroRNA-21 has the highest gene score (Figure 6C) and pathway score (Figure 6D) indicating that it targets most number of pathway genes and plays a noteworthy role in various pathway dysregulations as studied by researchers.

### Utility of miRwayDB

- On the basis of the information stored in miRwayDB, researchers can generate novel hypothesis to identify new correlation between miRNA-pathways and diseases.
- miRwayDB provides miRNA, gene and pathway scores. These scores can be used to evaluate the importance of miRNA regulated pathways in various pathophysiological conditions especially when considering the epidemiologically significant critical clinical conditions.
- The miRNA-gene-pathway-disease relationship information stored in the database can be used as the basis for integrated analysis of other biological levels such as biological processes, transcription factors, miRNA-gene polymorphisms and so on.
- Some studies provide more than one miRNA-pathway (or miRNA-gene or miRNA-disease) relationships as final results. Datasets stored in miRwayDB can be used as standards to validate these results.
- Datasets stored in miRwayDB can be used to develop different types of computational models.

### Comparison to other related resources

With rapid progress of research on dysregulated miRNAs and their role in various diseases, several miRNA-disease association databases were developed, such as miR2Disease (28), PhenomiR (29) and HMDD (27). These databases mainly focused on dyregulation of miRNA expression in various diseases. In contrast to these resources, miRwayDB is the first database that provides dysregulated pathway data in addition to miRNA-disease information. miRPathDB (24) is another database which provides information about ‘miRNA-gene-pathway’ but the human ‘disease’ component is missing. In comparison to miRPathDB, our database provides experimentally confirmed ‘miRNA-gene-pathway-disease’ information.

Among the several miRNA-disease databases, HMDD v2.0 (27) provide two miRNA related metrics, disease spectrum width of a miRNA and miRNA spectrum width of a disease to assess the importance of miRNAs in human diseases. In miRwayDB database, we introduced pathway scores as well as gene and miRNA scores to evaluate the role of miRNA regulated pathways in various pathophysiological conditions.

## Conclusion

The role of miRNA function and dysfunction in many critical diseases indicate that restoration of miRNA expression may correct these disease conditions. To provide valuable information regarding role of miRNAs in dysregulated pathways, we have developed a manually curated miRwayDB database which records an exhaustive list of miRNA-pathway relationships for 76 disease conditions from 663 articles. miRwayDB is not only a resource for miRNA-gene-pathway-disease associations but it can also be used for other biomedical approaches such as validation of computational analysis, integrated analysis, prediction of computational model and so on. The miRwayDB database will be regularly updated by integrating more data from publications. We would also encourage authors of new articles to submit novel miRNA-pathway data of various critical illnesses and make the database more useful and comprehensive. We believe that miRwayDB will help medical researchers to design further experimental research in the future.

## Future perspective

In the future, we will expand the miRwayDB by integrating more data from literature.
